# Supplementary figures and images for: Control of stereocilia length during development of hair bundles
Source: PLoS Biol. 2023 Apr 3;21(4):e3001964. doi: 10.1371/journal.pbio.3001964 (PMC10101650; doi:10.1371/journal.pbio.3001964)

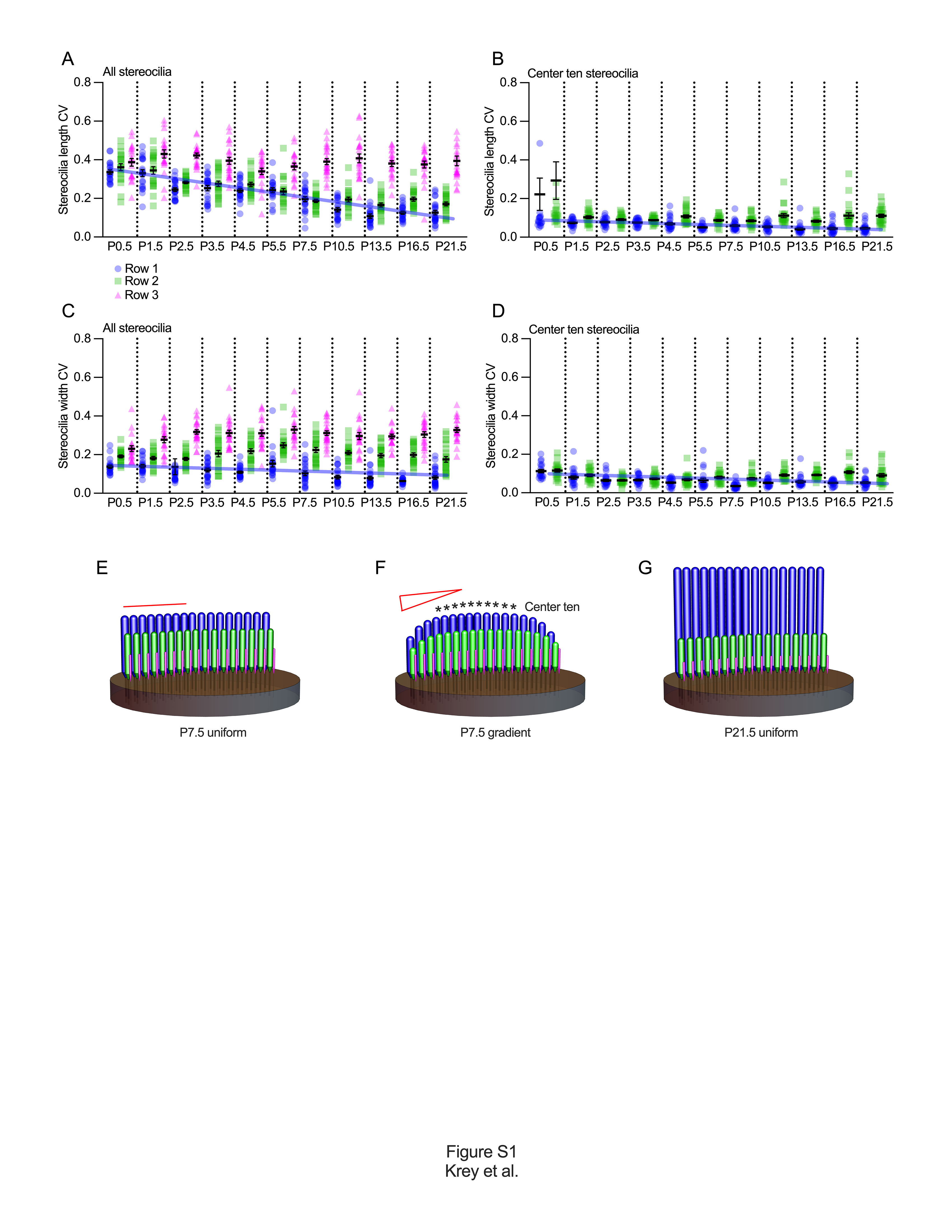

Supplement: S1 Fig — (A) CVs for stereocilia length from all stereocilia at indicated ages. Blue lines in panels A–D show developmental trends for row 1 length or width CV. (B) CVs for stereocilia length from center 10 stereocilia at indicated ages. P values from unpaired Student’s t tests comparing all row 1 stereocilia versus center 10 row 1 stereocilia: P0.5, 0.1861; P1.5–P21.5, <0.0001. P values from unpaired Student’s t tests comparing length CVs for all row 2 stereocilia versus center 10 row 2 stereocilia: P0.5, 0.4933; P1.5–P21.5, <0.0001. (C) CVs for stereocilia width from all stereocilia at indicated ages. (D) CVs for stereocilia width from center 10 stereocilia at indicated ages. P values from unpaired Student’s t tests comparing length CVs for all row 1 stereocilia versus center 10 row 1 stereocilia: P0.5, 0.0473; P1.5, <0.0001; P2.5, 0.0893; P3.5–P4.5, <0.0001; P5.5, 0.0001; P7.5, <0.0001; P10.5, 0.0038; P13.5, 0.0823; P16.5, 0.0126; P21.5, 0.118. P values from unpaired Student’s t tests comparing all row 2 stereocilia versus center 10 row 2 stereocilia: P0.5–P21.5, <0.0001. (E–G) Models of stereocilia arrangement. A peripheral-to-central gradient of stereocilia length for row 1 and row 2 was seen at P7.5 (F; red gradient symbol), rather than uniform lengths (E; red line). Stereocilia lengths largely equalized by P21.5 (G). The data underlying all the graphs shown in the figure can be found in figshare (https://doi.org/10.6084/m9.figshare.21632636.v2). (TIF) [file pbio.3001964.s001.tif]

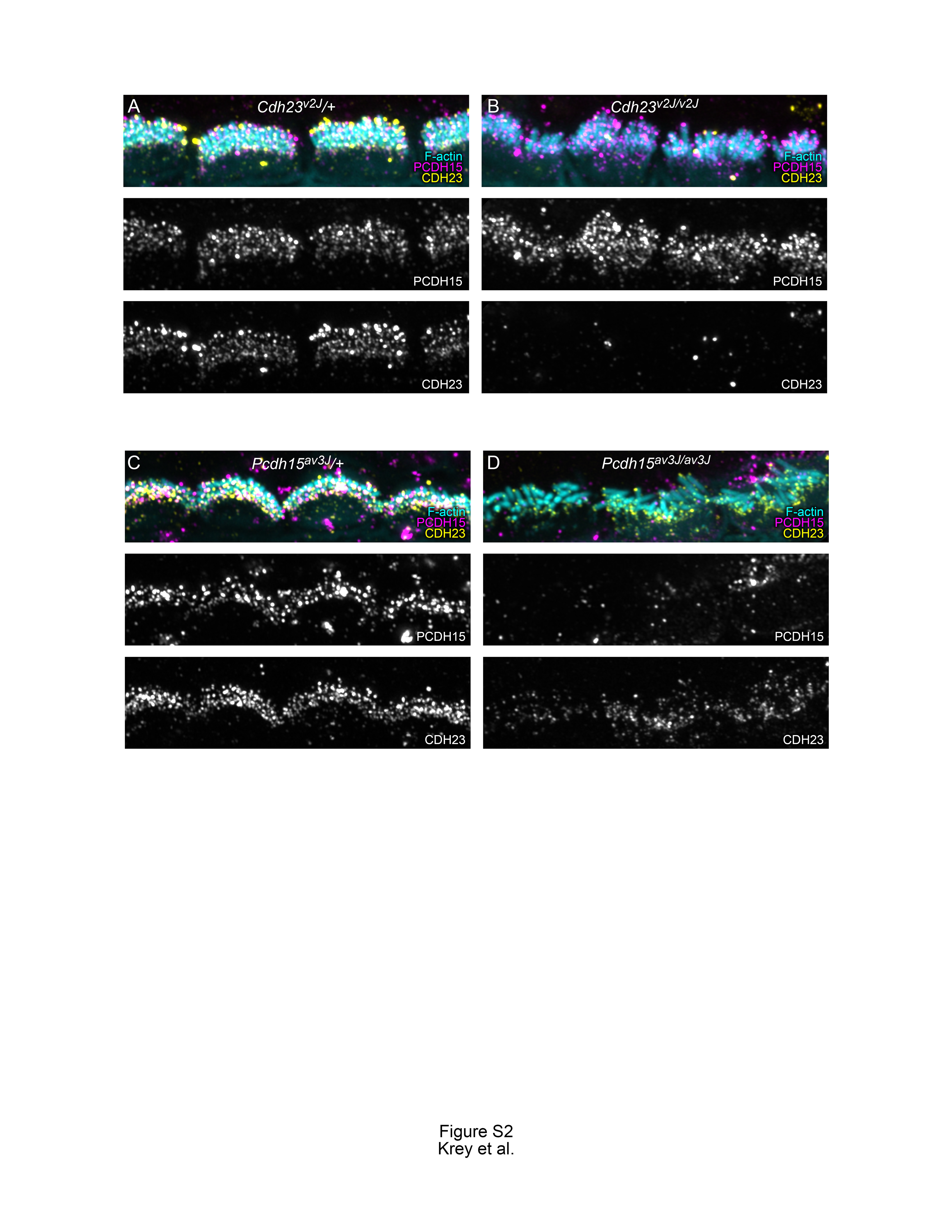

Supplement: S2 Fig — (A, B) CDH23 and PCDH15 localization in Cdh23v2J/+ (A) and Cdh23v2J/v3J (B) IHCs. (C, D) CDH23 and PCDH15 localization in Pcdh15av3J/+ (C) and Pcdh15av3J/av3J (D) IHCs. Panel widths: 30 μm. (TIF) [file pbio.3001964.s002.tif]

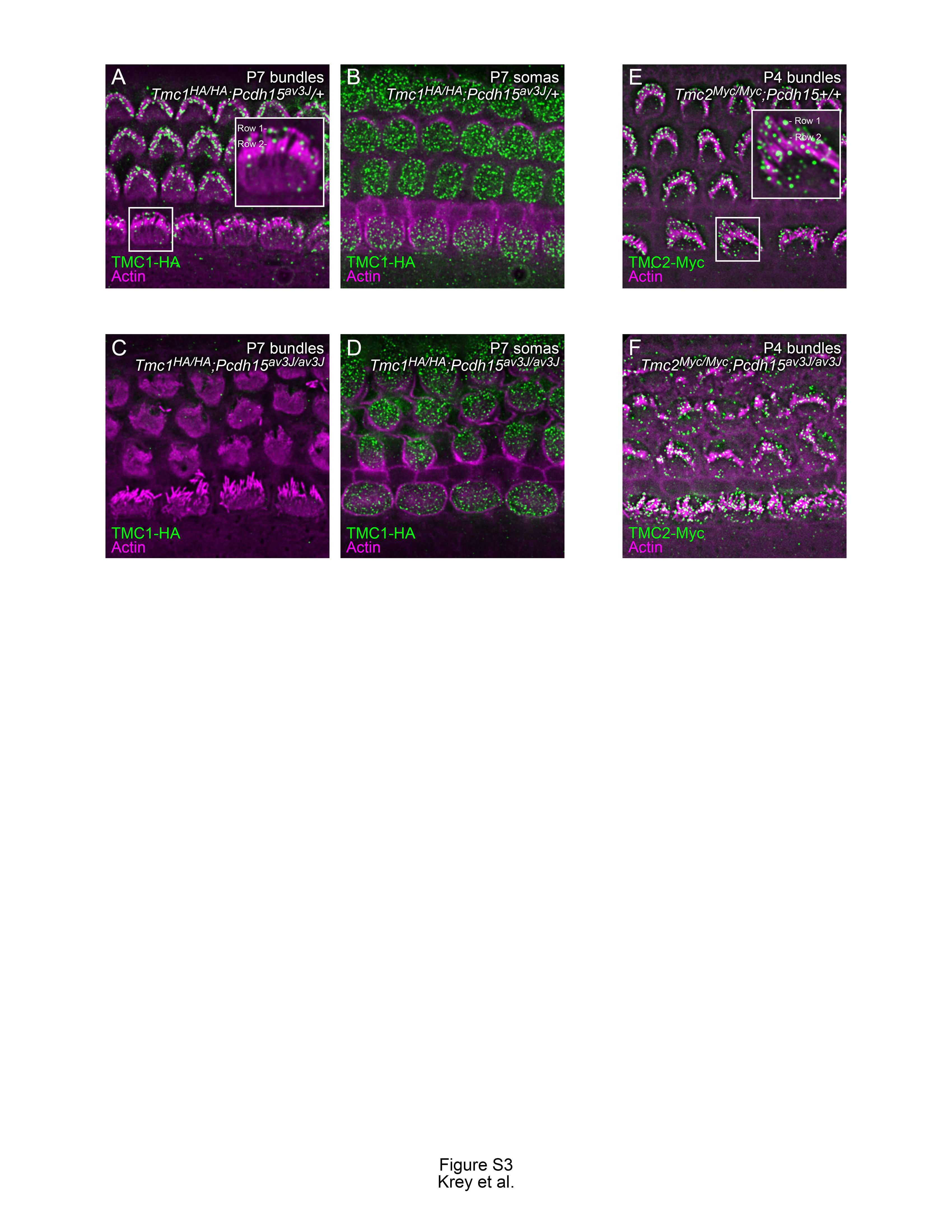

Supplement: S3 Fig — (A) TMC1-HA in P7 Tmc1HA/HA;Pcdh15av3J/+ cochlea at the hair-bundle level. Inset shows labeling in rows 1 and 2. (B) TMC1-HA in same P7 Tmc1HA/HA;Pcdh15av3J/+ cochlea at the soma level. (C) TMC1-HA in P7 Tmc1HA/HA;Pcdh15av3J/av3J cochlea (bundles). (D) TMC1-HA in same P7 Tmc1HA/HA;Pcdh15av3J/av3J cochlea (somas). (E) TMC2-Myc in P4 Tmc1Myc/Myc;Pcdh15av3J/+ cochlea (bundles). Inset shows labeling in rows 1 and 2. (F) TMC2-Myc in P4 Tmc1Myc/Myc;Pcdh15av3J/av3J cochlea (bundles). Panel widths: 35 μm (insets, 7 μm). (TIF) [file pbio.3001964.s003.tif]

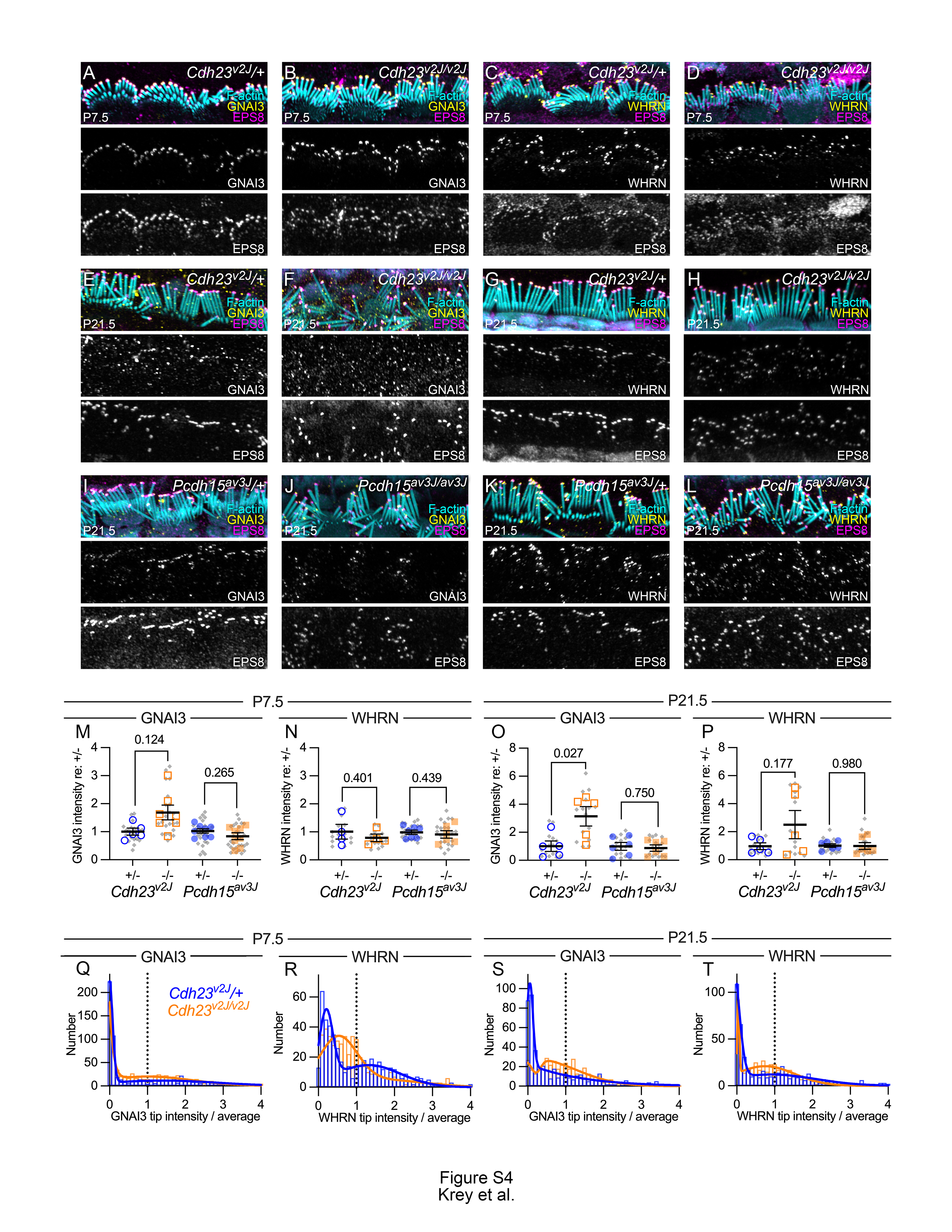

Supplement: S4 Fig — (A–L) Localization of row 1 complex proteins in Cdh23v2J and Pcdh15av3J heterozygotes and homozygotes IHCs. Panel widths: 30 μm. (A, B) GNAI3 and EPS8 in Cdh23v2J/+ and Cdh23v2J/v2J at P7.5. (C, D) WHRN and EPS8 in Cdh23v2J/+ and Cdh23v2J/v2J at P7.5. (E, F) GNAI3 and EPS8 in Cdh23v2J/+ and Cdh23v2J/v2J at P21.5. (G, H) WHRN and EPS8 in Cdh23v2J/+ and Cdh23v2J/v2J at P21.5. (I, J) GNAI3 and EPS8 in Pcdh15av3J/+ and Pcdh15av3J/av3J at P21.5. (K, L) WHRN and EPS8 in Pcdh15av3J/+ and Pcdh15av3J/av3J at P21.5. (M–P) GNAI3 and WHRN normalized fluorescence average intensity per hair bundle for all measured stereocilia (rows 1 and 2) for Cdh23v2J and Pcdh15av3J IHCs at P7.5 and P21.5, respectively. Intensities were normalized to the heterozygote average for each genotype pair. Plotting and statistical testing were as in Fig 3. (Q–T) Frequency distribution of GNAI3 and WHRN tip intensity in Cdh23v2J hair cells at P7.5 and P21.5. The data underlying all the graphs shown in the figure can be found in figshare (https://doi.org/10.6084/m9.figshare.21632636.v2). (TIF) [file pbio.3001964.s004.tif]

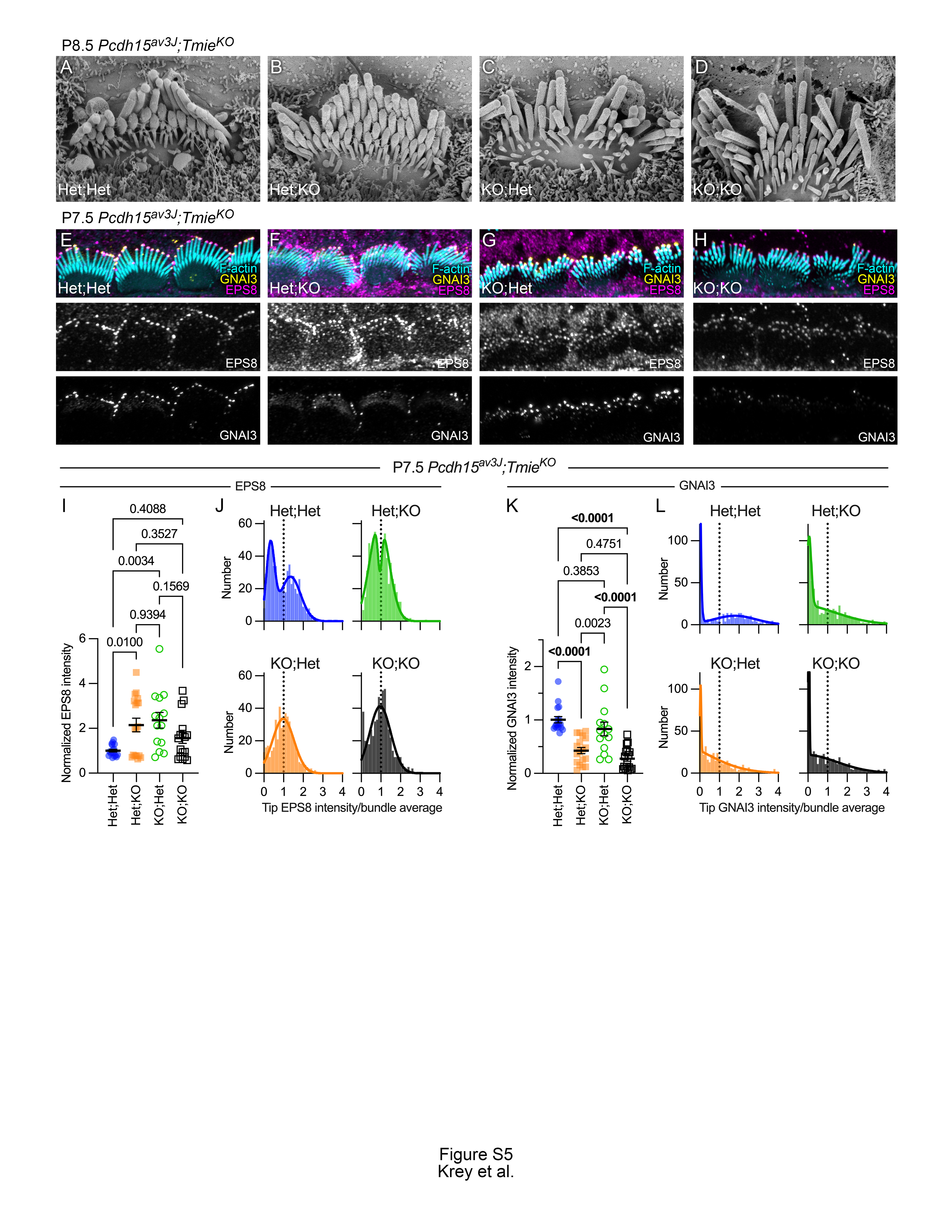

Supplement: S5 Fig — (A–D) Scanning electron micrographs showing P8.5 IHC hair bundles from Pcdh15av3J/+;TmieKO/+ (Het;Het) Pcdh15av3J/+;TmieKO/KO (Het;KO), Pcdh15av3J/av3J;TmieKO/+ (KO;Het), and Pcdh15av3J/av3J;TmieKO/KO (KO;KO) cochleas. (E–H) Localization of GNAI3 and EPS8 in IHCs of Pcdh15av3J;TmieKO genotypes at P7.5. (I) EPS8 fluorescence average intensity per bundle for all measured stereocilia in bundles of each genotype. (J) Frequency distribution of EPS8 tip intensity in bundles of each genotype. Het;Het and Het;KO distributions were fit with double Gaussians; KO;Het and KO;KO distributions were fit with single Gaussians. (K) GNAI3 fluorescence average intensity per bundle for all measured stereocilia in bundles of each genotype. (L) Frequency distribution of GNAI3 tip intensity in bundles of each genotype. Distributions from each genotype were fit with double Gaussians. For statistical comparisons in I and K, we used ordinary one-way ANOVA tests with the Tukey correction. The data underlying all the graphs shown in the figure can be found in figshare (https://doi.org/10.6084/m9.figshare.21632636.v2). (TIF) [file pbio.3001964.s005.tif]
