# Supplementary material for: Control of stereocilia length during development of hair bundles
Source: PLoS Biol. 2023 Apr 3;21(4):e3001964. doi: 10.1371/journal.pbio.3001964 (PMC10101650; doi:10.1371/journal.pbio.3001964)
Supplement: Attachment — Submitted filename: Summary suggestion.pdf [file pbio.3001964.s006.pdf]

“tip-link” mutants

transduction mutants

loss of
transient links
kinocilial links

loss of transduction
(Ca++)

loss of transduction
(Ca++)

loss of transduction
proteins at (row 2-3) tips

disarrayed placement

uneven length
inside a row

reduced row 2 width
reduced free barbed ends row 2
defective pruning

Increase of EPS8 in row 2 tips

reduced GPSM2-GNAI3 at row 1

↓

reduced row 1 height

↓

supernumerary rows
