# Supplementary material for: Control of stereocilia length during development of hair bundles
Source: PLoS Biol. 2023 Apr 3;21(4):e3001964. doi: 10.1371/journal.pbio.3001964 (PMC10101650; doi:10.1371/journal.pbio.3001964)
Supplement: Attachment — Submitted filename: PLOS Biology response to reviewers 2023-02-21a.docx [file pbio.3001964.s008.docx]

Editor:

In light of the reviews, which you will find at the end of this email, we are pleased to offer you the opportunity to address the comments raised by the reviewers in a revision. While the reviewers provide quite a number of suggestions for improvements to the manuscript, much of this relates to improving the interpretations and explanations so we anticipate the revision should not take you very long. When the revision comes back in, we will assess your revised manuscript and your response to the reviewers' comments with our Academic Editor aiming to avoid further rounds of peer-review, although we cannot rule out a need to consult with the reviewers when the revision comes back in.

Reviewer #1:

Reviewer #1: The development of the precisely organized mechanosensory stereocilia bundles in the inner ear hair cells is still a puzzling problem of cell biology. This manuscript provides comprehensive and meticulous quantification of the stereocilia growth in postnatal development in wildtype mice and mouse mutants lacking either only mechanotransduction or also tip links gating the mechanotransduction channels. These developmental data are correlated with the expression of the proteins that are known to be essential for the development of the proper shape of the hair cell bundle. Even though a similar study has been recently published by the authors (Krey et al., Curr. Biol., 2020), the extended set of data provided in the current manuscript reveal new roles of tip link proteins and allow building the molecular model of hair bundle development.

Specific comments:

Line 110: Could the exact location of the "apical IHCs" be better specified here (not only in Methods)?

We changed: “*We determined dimensions of stereocilia F-actin cores, labeled with fluorescent phalloidin, which were imaged in apical IHCs of C57BL/6 mice at precise times during early postnatal development.”*

To read: “*We determined dimensions of stereocilia F-actin cores, labeled with fluorescent phalloidin, which were imaged in ~~apical~~ IHCs of C57BL/6 mice at precise times during early postnatal development****. We used IHCs from the basal half of the apical turn of the cochlea (17-33% of cochlear length)****.”*

Legend to Fig. 1H: Fit line for the row 2 data peaked at ~P7.5 and then decline. This cannot be a "fit with an exponential climb".

The reviewer is certainly right. Changed to: *“In the panel, data for row 1 and for row 2 from P0.5-P7.5 were fit with an exponential climb; data for 2 from P7.5-P21.5 were fit with an exponential climb.”*

Fig. S1E is confusing - the hair bundle is never "flat headed" at P7.5 as it is depicted in this cartoon. Maybe, the authors meant to show the shape of the bundle at P0.5? Then, still, the panel A data demonstrate significant variability in the lengths of stereocilia within the row 1. Please, explain or correct.

We agree that the bundle is never flat-headed at P7.5—that is the point of the figure. One might think naively that bundle was shaped like S1E, but the data suggest variability. Our observations are that the bundle is more like S1F. The text notes: *“A peripheral-to-central gradient of stereocilia length for row 1 and row 2 was seen at P7.5 (F; red gradient symbol), rather than uniform lengths (E; red line).”*

Line 195: Data in Fig. 1N seem to contradict the data in Fig. S1B. Fig. S1B shows that the variability of stereocilia lengths within row 2 is larger than that in row 1 from ~P4.5 onward. In contrast, panel N in the main Fig. 1 shows no difference in the length variability between the rows. The row 1 vs 2 difference might be an important observation for the mechanisms of row 2 regulation and, therefore, it is worth clarifying.

There is no contradiction. The CV (in S1B) shows the **relative** error; with the same absolute error (what is plotted in Fig 1N), row 2 will show a greater relative error because the length (the divisor for CV) is less.

Lines 205-206: I think the statement "…while phenotypes specific for tip-link mutants arise from the absence of the links themselves" is valid only partially. The correct statement would be "…while phenotypes specific for tip-link mutants arise from the absence of the links themselves and/or the lack of signaling from intracellular domains of PCDH15 and/or CDH23".

Corrected to: *“Phenotypes shared by tip-link and transduction mutants most likely result from the loss of transduction, while phenotypes specific for tip-link mutants arise from the absence of the links themselves* ***or of signaling from their intracellular domains****.”*

Legend to Fig. 2: "Some links were still present in both Cdh23v2J/v2J and Pcdh15av3j/av3J bundles, but were not located at the apices of a stereocilia tips". This is misleading since there are plenty of tip link-like structures in the panel Eii (right in the middle of the bundle). They seem to be absent in Pcdh15av3j/av3J mutants, which may be consistent with the different roles of PCDH15 and CDH23 in formation of the transient links. In any case, the quoted statement above requires either clarification or the exact definition of "the apices of a stereocilia tips" and proper quantification.

The reviewer is correct. In reality, one can make the point instead that in the two mutant lines, there are no links associated with beveled tips.

Changed to: *“Some links were still present in both Cdh23^v2J/v2J^ and Pcdh15^av3j/av3J^ bundles, but were not associated with beveled stereocilia tips.”*

Lines 232-233: "…consistent with tip links causing membrane tenting and increased actin polymerization". Well, beveled row 2 tips are also observed after blocking transduction channels and retraction of row 2 stereocilia (Velez-Ortega et al., 2017). Thus, it is not clear whether the beveled row 2 tips in TmieKO/KO IHCs are caused by the increased actin polymerization around the lower end of the tip link or by increased disassembly of the peripheral actin filaments (which would be more consistent with the thinning of row 2 stereocilia reported in Fig. 4E). In the latter case, mechanical tension or some signaling associated with PCDH15 may protect the filaments surrounding the lower end of the tip link.

The reviewer makes a good point. Changed *“increased”* to *“altered”* to encompass several possibilities.

Fig. 3: Presumably, all mutant hair cells (Cdh23v2J/v2J, Pcdh15av3J/av3J, and TmieKO/KO) develop without functional mechanotransduction. Yet, only TmieKO/KO hair cells (as well as other transduction mutants, see Krey at all., 2020) show the mis-localization of the row 1 complex and retarded growth of row 1 stereocilia at P21.5. How is it possible, if TMC1 is also missing in the tip link mutants (at least in Pcdh15av3J/av3J mutants, Fig. S3C)?

The reviewer points out a fascinating paradox that we have not yet resolved. Fig S3C only shows that TMC1 does not go into stereocilia in Pcdh15 mutants, but it is still expressed. Our working model is that the transduction complex regulates GPSM2/GNAI3 entry into row 1 tips by exerting its action at the base of the stereocilia; we presume that in tip link mutants, the transduction complex is still located at the place where it regulates GPSM2/GNAI3 entry.

Fig. 5, panels D & F: I cannot understand why the coefficients of variation for delta lengths (bottom graphs) are similar between wild type and Cdh23v2J/v2J and Pcdh15av3J/av3J mutants, while the spread of the same raw data (upper panels) is visually different.

This difference (and the contrast with length measurements, as in Fig. 3) is because we are measuring a small length difference. The top panel (Δ length) represents the **average** difference between adjacent stereocilia when comparing multiple bundles.

However, because the differences in length (~1 µm) are relatively small compared to what we assume is the intrinsic measurement error, the CV values in the top panel are high (compare to the CVs in Fig. 3). If the measurement error was smaller, we would expect to see the spread of the data show up in the CV plots.

In general, it is very hard to understand this convoluted way of presenting relatively simple differences in the shapes of different bundles. Why the same data cannot be presented as a collection of graphs, which would show the length of individual stereocilium (Y-axis) relative to ts position within a row (X-axis)? Two-way ANOVA would be useful for statistical analysis, while the source of within-the-row variations would become visually obvious.

In Krey et al. (2020), Fig. S2 shows plots similar to the ones that the reviewer is asking for. These plots show the difference in height across the bundle, and the CV measurements show the increased variability in length of stereocilia in Tmie mutants.

There are some advantages in using a plot like this, as it shows variation across the bundle, but there are also advantages in plotting as we have in the present figure. In particular, it allows us to condense the results from each mutant into a relatively uncomplicated plot.

The advantage of this analysis is that it separates the variation of stereocilia length at the level of an individual hair bundle (which you get from the CV measurements—**averages of CVs** across many bundles) from the variation of stereocilia length from bundle to bundle (**average length** across many bundles).

Where this analysis is particularly useful is when the average stereocilia length across many bundles does not change (e.g., row 2 in Fig. 3Ji) but there is considerable variability in each individual bundle (e.g., Fig. 3Jii). This result is consistent with the idea that there is a fixed amount of actin building blocks; even though there is considerable stereocilium-to-stereocilium variability within hair bundles, their average lengths are the same.

Lines 340-341: "…perhaps because the minimal staircase prevented tip links from sufficiently tenting membranes off the tips of the shorter rows". Alternatively, the tenting may be harder to generate when the link pulls the membrane at the wrong place, on the side of a stereocilium instead of the tip.

We think those two options are the same thing.

Fig. 6: Do the double mutant IHCs loose all their links between stereocilia? SEM images and/or link quantification would be helpful here.

Because of breeding timing, we did not have SEM of these double mutants complete at time of submission. We do now, and have included those SEM data—two examples of each of the three genotypes that we highlighted.

The DKOs do not lose all their links, but neither did the Pcdh15 single mutants. We make this point with the following lines: *“Links near stereocilia tips were present not only in shaker 2 KO bundles (Fig 6B), as expected, but also in Pcdh15;Myo15a DKO bundles (Fig 6C). As in Pcdh15^av3J/av3J^ bundles (Fig 2F), remaining ankle links and CDH23-CDH23 links may maintain stereocilia interactions in Pcdh15;Myo15a DKO bundles.”*

Fig. 8: This is great! Very nice piece of data.

It’s a nice confirmation of Tilney’s idea.

Line 628: You meant "…during stage III, when lengthening is arrested"?

That is what we meant to say. However, since our study focused on stages III and IV, we dropped this line as we have no data supporting the contention (see discussion below with reviewer #2).

Line 836: Could the authors provide SEM evidence for the tip links running "to the end of the track"? The links connecting the tips of the adjacent stereocilia in a column should be quite prominent.

It is impossible, of course, to see the actin tracks in SEM, and especially to see where the peripheral actin filaments adjacent to the membrane stop. That stereocilia tips are rounded and not square makes it clear that the filament structure must be this way, which has been experimentally confirmed (e.g., with cryo-EM tomography; see Song et al., 2020).

We realize that the reviewer is suggesting that we provide evidence for tip links stopping and building up at the end of the tracks; you might expect to see a ring of links where the stereocilia start rounding at their tips.

That said, the links in Myo15ash2/sh2 stereocilia do seem to be at this position, so they may be at the ends of their tracks there.

Nevertheless, we are less confident in this model for stereocilia elongation, so we have tempered our conclusions in this part of the Discussion: *"* ***Thus in one model****, actin polymerization proceeds as the membrane is tented, allowing stereocilia lengthening (40). During stage II, the shorter rows may lengthen simultaneously with row 1, but stereocilia in the shorter rows may be limited in their maximal length because the molecular motor pulling on the tip link would presumably run to the end of its track on the adjacent (taller) stereocilium."*

Lines 843-862: I would refrain from speculating on the mechanisms of resorption of supernumerary stereocilia. The manuscript data are focused on row 1 and 2 and formation of the staircase. There is only very limited amount of data regarding rows 3 and 4+ and, therefore, these speculations are not yet supported by the data.

Relatively few points are made about resorption. Moreover, the “building block availability” hypothesis is supported by the data and it does suggest appropriate speculation.

Lines 884-886: "More randomized allocation of row 1 complex members and the varying positions of stereocilia across the lateral-medial resource gradient may also create length variability". This statement seems contradicting the idea of the control of length variability through lateral PCDH15 and CDH23 links.

The discussion point is specifically about the *Cdh23^v2J/v2J^* and *Pcdh15^av3J/av3J^* mutants. To clarify this, the sentence was changed to read: *“More randomized allocation of row 1 complex members and the varying positions of stereocilia across the lateral-medial resource gradient may also create length variability* ***in Cdh23^v2J/v2J^ and Pcdh15^av3J/av3J^ mutants****.”*

Lines 904-906: Then, it should be possible to prevent stage III and force stereocilia to grow without widening by blocking the transduction channels. Is it true?

As we show in Krey et al. (2020), it’s not that simple. The stop to lengthening is associated with the appearance of transduction, as is the focus on widening rows 1 and especially 2. The rows still widen in transduction mutants, however—but all the rows widen relatively similarly.

Line 1078: Remove extra "E".

Done.

Reviewer #2:

The author's objective is to describe P7-P21 stereocilia development in IHCs at the cochlear apex and to investigate structural and molecular defects in 3 categories of mutants that were shown to affect elongation. This work was started in Krey 2020 [6], where focus was on transduction mutants and the role of Ca++ in shaping stereocilia dimensions.

Actually, P0-P21.

In this new work, the main difference is the inclusion of "tip-link" mutants (that in fact also disrupt transient lateral and kinocilial links), the use of an improved microscopy method (lattice SIM; allows for example to address elusive row 3 stereocilia), surface rendering and an (even more) exacting approach with more hair cells and stereocilia data points measured in a finer time course. As the work endeavors to sum up a lot of results and to serve as current reference in the field, it is fitting for a Methods and Resources article. These methods are further applied to an interesting budding question: how do mechanotransduction elements influence stereocilia morphogenesis? The work is very well executed and sets a high standard for rigorous and thorough methods, quantification, and data reporting.

The next challenge, however, is to present so many results, often disparate, as a coherent whole, and chiefly to direct the reader towards more notable new results that are solid while acknowledging and nuancing those that remain more uncertain. This aspect could be improved in a revised version that could perhaps be shorter as well. We feel that the unusually large body of data is challenging to navigate, hiding some gems while alternatively devoting much space to a few conclusions that are not necessarily fully supported by evidence here or in Krey 2020.

In short (further detailed below), we find that several claims about the behavior of the actual row 1 complex (GPSM2-GNAI3 and WHRN; see point B2 below) are not fully convincing, with caveats that call for language to be revised to better reflect evidence reported here, in Krey 2020, and in other referenced papers. Interestingly, this comment holds particularly true for the Introduction and Results, whereas by contrast the Discussion presents a fairer and more balanced summary of results in general, although with some degree of repetition. Discussion positions should be adopted in the rest of the manuscript, as readership is likely to focus chiefly on Intro and Results first.

The detailed comments below are made in hopes that they can help the authors improve the manuscript, notably A) better highlight some remarkable results and conclusions while B) avoid possibly hasty conclusions about the behavior of the row 1 complex. We are not requesting additional or revised experiments, except if the authors want to maintain currently less-well supported claims (see below)

We appreciate the remarkably detailed comments offered by this reviewer. The reviewer has noted some shortcomings in our interpretations and presentation that definitely need to be corrected. We do not agree with all of the reviewer's conclusions and proposed edits, but all of them have been considered very thoroughly. The input makes the manuscript stronger, and we are thankful for that.

**A) RESULTS THAT ARE UNDER-STATED**

**A1)** From the onset, the main expectation for this paper seems to be a close comparison of two categories of mutants that both abolish transduction, allowing the authors to tease apart the specific role of MET channel proteins or fibrous links from the process of transduction itself.

The “main expectation” for the paper is not just comparison of two types of mutants that abolish transduction. Instead, that comparison was just one of several tools we used to probe length regulation in apical IHCs; we also examined changing dimensions in B6 WT hair cells, as well as used shaker 2 mutants to examine the role of complexes that include MYO15A.

This is made explicit in the text early in the manuscript:

Abstract Line 30:"Use of the tip-link mutants also allowed us to distinguish the role of transduction from effects of transduction proteins themselves."

Line 204: "Phenotypes shared by tip-link and transduction mutants most likely result from the loss of transduction, while phenotypes specific for tip-link mutants arise from the absence of the links themselves.".

These are true statements, but they do not represent the only avenue we pursued.

Surprisingly, however, this angle is somewhat abandoned en route, even though remarkable and complete results are obtained and detailed on this front.

In short, this work shows that the two phenotypes are interestingly distinct from one another. "Tip-link" mutants have overall normal row 1 lengths and tip complex but variable lengths in each row and disarrayed stereocilia placement (packing issues), likely through lack of transient and kinocilial links. In contrast, transduction protein mutants have shorter row 1 lengths and reduced row 1 complex, but more even stereocilia lengths within rows; they also have a unique extra rows phenotype classic of “mature row 1” complex mutants (*Myo15a* etc.).

What appears to be shared are the following defects:

- row 2 width reduction (Fig. 4)

- increase in row 3-4 volume (Fig. 7), or a shared defect in pruning

- reduced free barbed ends in row 2 at P7 (Fig. 13)

This somewhat limited overlap is surprising and novel, and is thus expected to be at the core of the paper’s Discussion and summary diagram in Fig. 15 - but is not. To try to illustrate this point, we attached a rough diagram of what we would have expected to (also) see in a graphical summary.

The reviewer is correct that this limited overlap is surprising, but that does not mean it needs to be at the core of the paper’s Discussion and summary diagram. Instead, we suggest that the results presented here plus the outstanding previous work from many investigators puts us in place where we can have a more comprehensive view of stereocilia growth, especially during stages III and IV.

That said, while the proposed diagram serves a different purpose than Fig 15, it is indeed a very good idea. We made a table (Table 1) that serves the purpose suggested by the reviewer. We also added a new section to the Discussion, “***Transduction mutants and tip-link mutant have overlapping phenotypes***,” which compares the shared and distinct phenotypes and refers to Table 1.

For one, these results suggest that MET function itself (Ca++) plays a lesser role in stereocilia morphogenesis compared to the model proposed previously (Krey 2020, Velez-Ortega 2017). Instead, links (transient, kinocilial) are paramount yet distinct in function from the presence of transduction protein at row 2-3 tips.

We wouldn’t say that MET plays a lesser role, but rather that there are other key molecules controlling length and other stereocilia dimensions as well. Clearly MET function still plays a role in stereocilia width regulation and pruning, and given that changes in actin/building block distribution can ultimately affect availability for other types of growth, we would conclude that transduction itself plays as much of an overall role as the links do.

In Krey 2020, blocking MET at early stages with tubocurarine yielded extra stereocilia rows, yet largely normal row 1 complex enrichment. Consequently, extra rows appear to result from ablating MET function when transduction proteins are present, so why do "tip-link" mutants lack extraneous stereocilia rows despite lacking transduction? This conundrum deserves acknowledgement and discussion. It would also be interesting to speculate on the "other" role of transduction proteins besides enabling transduction.

We don't agree that tip link mutants *"lack extraneous stereocilia rows."* The tip link mutants do have extraneous thicker stereocilia, but they are just not organized into rows as in the other mutants. The pruning data show this (Fig. 7I-L).

**A2)** Other solid and novel results are discussed but may benefit from being further highlighted. The long Discussion seems to show some redundancy with some themes "echoed" back a few times - consolidating might be more effective (see also Minor points list).

These interesting and novel results are for example:

- First formalized report that peripheral stereocilia are initially shorter compared to central ones.

Krey et al. (2020) showed this directly (in Supplemental Figure 2), and it was also shown in a more limited way in Miller et al. (2021). We agree that this is the first place this observation is highlighted.

- Dynamic fit allowing to distinguish different cross-sectional area increase regimes (linear before P8, exponential afterwards)

The novelty really comes from the increased number of datapoints, allowing more precise fitting. We do not believe that this warrants special attention.

- Total stereocilia volume constant in all mutants. Interesting support for the idea that hair cell “building blocks” remain constant and thus stereocilia dimensions are defined by how building blocks are put together, not their availability.

We agree that this is an important observation, and it is woven into the Discussion in multiple places.

- Proposed mechanism to coordinate lengths in the same row (links; but kinocilial link contribution to packing issues for example is not highlighted)

We did spend considerable amounts of manuscript spacing describing and discussing this observation. For example, in the Discussion, the section titled “***Loss of CDH23 or PCDH15 leads to uncoordinated stereocilia lengthening***” (lines 724-773) is devoted to this mechanism.

- Absence of TMC1 (but not TMC2) in Pcdh15 KO

This is a relatively minor observation.

Free barbed ends largely lost at P21 but equally retained in the 3 mutant categories- is it bundle immaturity?

Correct, the decline at P21.5 (and maintenance in the mutants) is a novel result. We discuss the result in the text and connect it to CAZPB accumulation in row 2 by P21 (i.e., the absence of CAPZB accumulation in the mutants).

- Missing discussion of compound mutants in Figure 6 and S5. Interesting but significance remains unclear.

We focused more attention on results from *Pcdh15;Myo15a* DKOs. We noted in Results: *“These results suggest that row 1 complex proteins are required to elongate stereocilia before PCDH15-dependent links can help refine bundle architecture.”* In addition, we have a paragraph in the Discussion addressing the significance of the results.

For *Pcdh15;Tmie*, we noted in the Results: *“Mutations in Pcdh15 thus did not suppress transduction-mutant phenotypes, nor did mutations in Tmie suppress phenotypes seen in tip-link mutants, indicating a lack of genetic interaction for these phenotypes.”*

B) OVERSTATED CONCLUSIONS

**B1) Conclusions about "stereocilia development" are strictly based on select stages of IHCs at the cochlear apex.**

P0.5-P21.5 IHC behavior at the cochlear apex, in both normal and mutant contexts, may not seamlessly transpose to earlier stages, other cochlear positions, or to outer hair cells. For example, could the sharp increase of row 1 length at P8.5, or the transition between linear and exponential cross-sectional areas also at P8.5 be specific to the apex? The Tilney model of phase I-IV would greatly benefit from having mouse stages associated not only for the apex, but also the cochlear base to help translate these results to other contexts.

For example, is row 1 length at the cochlear base stable during stage III as reported here at the apex? It may instead be reduced during phase III to reach the short statures characteristic of this region?

The row 1 complex is prominent in OHCs too starting at earlier embryonic stages, and it may behave differently than IHCs at the apex in this context.

We certainly agree that extending this analysis to OHCs, as well as to mid and basal IHCs and OHCs, will be important for the future—we have indeed already begun this analysis. That said, we think that the reviewer would agree that keeping the present analysis confined to one type of cell at one location is important—the size of this manuscript could balloon even farther than its present (very) large size.

We do realize that the reviewer’s point is to be careful to restrict our conclusions to apical IHCs. That is fair. We have concluded the Discussion by making this point:

*“While results here are from apical inner hair cells, development of hair bundles from apical OHCs, as those of mid and basal IHCs and OHCs, may also follow the specific stages described here. This scheme was initially developed to describe bundle development in the chicken cochlea (1), and it has proved applicable to mammalian hair cells. Nevertheless, we expect that other cochlear hair cells in the mouse will regulate these stages at different times and to different degrees; differential regulation in turn will produce bundles with distinct stereocilia lengths and widths, as is seen across the cochlea. In the future, we will more broadly apply our quantitative approach to bundle development, so successful for apical hair cells, to determine the developmental principles that apply to all bundles.”*

We note that we have already begun to carry out this analysis with other cochlear hair cells, but it will take some time to complete.

Finally, we note that the previous paper (Krey et al., 2020) has limited length and width data for mid and basal IHCs. Our conclusions there were:

*"As expected because of basal-to-apical developmental progression, row 1 lengthening and row 2 shortening took place earlier in mid- and basal-cochlea regions (Figures S1I and S1K)."*

and:

*"Row 2 widening occurred earlier in mid- and basal-cochlea regions (Figures S1J and S1L)."*

**B2) Assuming that EPS8 (and MYO15A) are part of a "row 1 complex" during development**

Before P7.5 at least, if not later, EPS8 and MYO15A are clearly distributed in all rows, unlike GPSM2, GNAI3 and WHRN. This was reported by others, in Krey 2020, and is in fact even shown here [Fig 9B,F Fig 10C, G etc., Fig11D, H, L]. However this distinction is understated in the text of the Introduction and Results. Grouping EPS8-MYO15A and GPSM2-GNAI-WHRN together is too simplistic, especially for this rigorous work focusing on developmental stages where these proteins have distinct row distribution patterns.

We certainly understand this point, and agree that it is important to distinguish actions of EPS8/MYO15A alone from the whole “row 1 complex” aka “elongation complex” (all five proteins).

Of note, the fact that GPSM2-GNAI3 but not MYO15A-EPS8 is reduced at row 1 tips in transduction mutants in this work is an independent clue that these proteins form in fact distinct modules.

Yes, that is definitely true.

Locations where this issue is problematic:

Line 50: "A complex of GPSM2 and GNAI3, coupled to MYO15A and EPS8 by WHRN, catalyzes lengthening of stereocilia beyond about a micrometer [7–9]; this complex concentrates exclusively in row 1.".

In fact EPS8 and MYO15A are prominent in all rows notably row 2 until at least P7. This sentence makes a definitive statement that only applies to adults and should be edited.

Agreed. Edited sentence to read: *"A complex of GPSM2 and GNAI3, coupled to MYO15A and EPS8 by WHRN, catalyzes lengthening of stereocilia beyond about a micrometer* ***in postnatal stereocilia development*** *(7–9). This complex concentrates exclusively in row 1* ***after P7*** *and hence is referred to here as the row 1 tip complex."*

Line 443: "As in C57BL/6 stereocilia, in heterozygotes from mutant IHCs, GNAI3, GPSM2, and EPS8 were concentrated at tips of row 1 stereocilia (Fig 10A-D, I-L)."

This is inexact, or at least misleading, since EPS8 is well visible at row 1 unlike the other proteins.

We assume the reviewer means that *"EPS8 is well visible at row* ***2****…"* Our sentence is correct as written. Even though EPS8 is visible at row 2 (e.g., Fig. 10A), it is still concentrated at row 1 tips.

Line 446 “..but EPS8 was seen at all stereocilia tips in similar amounts”.

This formulation indirectly recognizes that EPS8 is normally at row 2 tips but in lower amounts, emphasizing the need to present it as such in controls before underscoring uniformity in mutants.

No, that formulation does not recognize that EPS8 is at low levels in row 2 tips. All the phrase says is that in *Pcdh15* mutants, row 1 and row 2 tips have similar amounts of EPS8. That is not true in controls, where EPS8 in row 1 > EPS8 in row 2.

Figure 9. should point out row number to show that EPS8 is in all rows. Fig. 9B shows EPS8 at row 2 and so does Fig. 9F, P7.5. This could be made more clear in the images.

Done. We have added callouts and arrows to indicate row 1 and row 2 in Fig. 9A-F.

It is notable that in Fig. 9, the next stage investigated after P7.5 is P15.5 where indeed EPS8 is very low at row 2. This minimizes the fact that EPS8 is enriched at row 2 (and 3+) from embryogenesis until probably ~P10 (Fig. 9I).

We disagree—the data are clear in Fig. 9I showing that EPS8 stays high in rows 1 and 2 at least until P10.5. We don't have space to show example panels for all ages.

All through Fig 10, EPS8 at row 2 is quite obvious at P7.5 as well, and could be pointed as such. Best example in C, G but visible in other panels too.

We also added callouts and arrows to indicate row 1 and row 2 in heterozygote panels in Fig. 10.

On the quantitative side, frequency distribution representations in Fig. 11 clearly show substantial EPS8 enrichment at row 2 at P7.5 and possibly later (Fig. 11D, H, L; two bumps of tip intensity/average values).

Agreed. We added the following text: *"…two prominent peaks were detected in Cdh23^v2J^/+ or Pcdh15^av3J^/+ controls at P7.5, consistent with high levels of EPS8 located at row 1 tips and lower levels present at row 2 (blue data in Fig 11D, F)."*

Lines 486 and 499, the text refer to "two prominent peaks" and " two well separated peaks", respectively, without ever relating this observation to significant EPS8 signal at row 2 in controls.

See above.

Line 465: "Quantitation of row 1 tip complex proteins allowed us to determine the distribution of these proteins between row 1 and 2 stereocilia more thoroughly (Fig 11-12; S4 Fig)."

The cited figures include P7.5 where in fact EPS8 is notably enriched at row 2 in controls.

Line 484: ”Frequency distribution plots, using normalized EPS8 intensities (signal at each tip divided by the total signal for all row 1 and 2 stereocilia measured in the hair bundle), revealed how EPS8 was uniformly targeted to stereocilia tips in tip-link mutants”.

Same comment as above line 446. This sentence makes conspicuous that the normal distribution of EPS8 in controls was actually not accurately described in the first place.

See above.

We also added the following line to the Introduction: *“MYO15A and EPS8 are found together at all stereocilia tips during early postnatal development, and likely control the initial lengthening of stereocilia (7).”*

**B3) Assuming GPSM2-GNAI3 is active at stage II and reduced at stage III (before an obvious increase at stage IV)**

This manuscript does not show stage II results, if we assume from Figure 1 that P0 apex is already stage III. The model proposed in Figure 15E states that GPSM2 is present and required for stage II elongation. Indeed, P0 IHCs halfway through the basal turn were reported to have shorter row 1 stereocilia already (Tarchini 2016), and if previous GPSM2 activity at this stage and position qualifies as stage II (and not already as stage III ?) this conclusion is justified.

Yes, we do not show stage II results. We do not have direct evidence in the present manuscript showing that GPSM2 triggers elongation in stage II, and so we have removed speculation about GPSM2 in stage II. We agree that Tarchini et al. (2016) shows clearly that in basal hair cells, *Gpsm2* KO row 1 stereocilia are already shorter than control stereocilia. Moreover, they continue shortening at least until P6 (Tarchini et al., 2016). At P0, however, basal hair cells are well into stage III (see Krey et al., 2020). Thus the Tarchini data do not distinguish between model A—GPSM2 causes stereocilia growth in stage II—and model B—something else causes stereocilia growth in stage II, and then GPSM2 stabilizes growth in stage III (during length arrest and widening). More evidence supports model A, we agree, but model B cannot yet be ruled out.

However, a reduction of GPSM2-GNAI3 or WHRN at row 1 tips after P0 and before P7 was not reported before, including in Krey 2020. We would argue it is not demonstrated here either. Krey 2020 showed a steady increase during stage III (Krey 2020; Fig. 3A-B), with clear GNAI3 enrichment at P2.5 and P4.5 at the cochlear apex. The authors would need to analyze earlier stages at the stage II-III transition in more details to make this claim (possibly including the cochlear base to assess how broadly applicable conclusions are). Is GPSM2-GNAI3 actually temporarily reduced at tips between stage II and IV? GPSM2-GNAI3 can be detected at P0 at the base in IHCs already, and would need to be reduced there by ~P2-P4 to claim a reduction during phase III.

We agree, see above.

Another issue with the model proposed here is that previous publications show GPSM2 as closely enriched with GNAI3 at ~P2-P4 already (stage III), so it is surprising that GPSM2 would be downregulated at tips (more so than GNAI3) to then peak again during stage IV, but in a delayed manner compared to GNAI3 .

In apical hair cells, we find that GPSM2 and GNAI3 show a similar rising pattern of expression from P0.5 to P7.5 and beyond. We agree, we present no evidence here for downregulation of GPSM2 (nor for GPSM2 expression during stage II).

An alternative explanation to the results in Figure 9 could be that the GNAI3 antibody used in this study is more sensitive than the GPSM2 antibody, picking up signal at tips more efficiently at P7.5. Or, the normalization process could possibly explain different peak times? See line 441-442, normalization method was different for GPSM2 and GNAI3.

We stand by our results here. While it is true that the GNAI3 antibody is more sensitive than the GPSM2 antibody, we are comparing within antibodies (looking at the profile over time) rather than between antibodies. If we underestimated GPSM2 at early time points, the peak in expression would not change.

The line 441-442 comment is not relevant, as we arbitrarily chose a timepoint for normalization. Choosing a different timepoint would not affect the pattern we see.

Locations where this issue is problematic:

Abstract Line 23 **"**Tip proteins that determine row 1 lengthening did not accumulate simultaneously during stages III and IV; while the actin-bundling protein EPS8 peaked at the end of stage III, GNAI3 peaked several days later—in early stage IV—and GPSM2 peaked near the end of stage IV."

Line 98: "Here we defined with higher precision the changes in stereocilia dimensions during postnatal development of apical IHCs from postnatal day 0.5 (P0.5) to P21.5."

Line 23 is accurate.

Line 98 is accurate.

As mentioned above, protein labeling at P0-P7 is not tested with the same precision as P7 and older in mutant contexts, with fewer images and measurements (Fig. 9-11).

Yes, this is true; we do not report here protein labeling in mutant hair cells earlier than P7.5. This still does not change the accuracy of line 98 since it refers to dimension measurements, not protein labeling. Line 23 is still accurate since we are describing what occurs in a wild type context, not a mutant one.

Line 427: “GNAI3 was relatively low during stage III, but increased to a peak at P10.5, near the beginning of stage IV”

This text does not capture well the graph results for GNAI3 in Fig. 9J. In fact, GNAI3 increases mostly during stage III. Krey 2020 reported similar results, with a large increase before P7.5.

We agree. The sentence was changed to: *"GNAI3 increased throughout stage III; it peaked at P10.5, soon after stage IV begins (Fig 9J)."*

Line 430: "Our results showing sequential accumulation of EPS8, then GNAI3, and finally GPSM2 at stereocilia tips are not consistent with the earlier suggestion that the row 1 tip complex assembles simultaneously [9]."

This sentence needs to be removed or heavily edited. First, see comment B2 above about the invalid "row 1 complex" to describe 5-protein complex during development. Second, the previous work cited (Tadenev 2019) actually already showed that EPS8 (and MYO15A) are detected at tips in all HCs before GPSM2-GNAI3, so this is not an accurate citation of the publication. Third, if Tadenev 2019 indeed proposed simultaneous enrichment of GPSM2-GNAI3, so did in fact the author's previous paper Krey 2020: “GPSM2 showed a nearly identical labeling pattern to that of GNAI3 (Figures S4A and S4B)."

We have revised the original conclusions that were in the Krey et al. (2020) paper by carrying out much more exhaustive quantitation in the present paper. We agree that the Tadenev et al. (2019) paper showed EPS8/MYO15A at all tips before GPSM2/GNAI3.

We edited the sentence to read: *"Our results showing sequential accumulation of EPS8, then GNAI3, and finally GPSM2 at stereocilia tips are not consistent with the earlier suggestion that an extended MYO15A-EPS8-WHRN-GPSM2-GNAI3 complex assembles near the stereocilia base and then moves to row 1 tips (7)."*

The underlined section is a paraphrase from the Tadenev et al. (2019) paper; we were just pointing out that we come to a different conclusion here. (And a different conclusion than we made in Krey et al., 2019.)

This sentence also disregards that conclusions in Tadenev 2019 were made also including OHCs where GPSM2-GNAI3 are heavily enriched at tips during embryogenesis already; earlier and at higher levels than in IHCs. This is bringing back point B1 about how universal or not the dynamic changes observed in IHCs at the cochlear apex are.

Until OHCs and mid- to basal hair cells are studied in detail, we agree that our results cannot be considered to be universal. That said, we are more comprehensive here about the progression of development in a single hair cell type—the apical OHC—and that approach is definitely revealing.

We do point out that quantifying protein expression across developmental time points is quite revealing, as what appears to be high levels of expression at one time point may actually not be that high when it is compared to other time points. Our future investigation of OHCs and mid- to basal hair cells will include quantitative measurements of proteins—like those in the row 1 complex—across developmental time

Line 823: "GPSM2 and GNAI3 levels are low during stage III widening".

Same comment as above. This work and Krey 2020 in fact show increased amounts from P0.5, which does not seem compatible with transient reduction at stage III.

We agree that there are no data supporting the idea of a transient reduction during stage III. There are also no compelling data for high levels of GPSM2/GNAI3 during stage II in apical IHCs. Accordingly we have edited this and the adjacent sentence to read: *" While EPS8 levels are high across stages III and IV, GPSM2 and GNAI3 levels are low at the beginning of stage III widening. As stage III progresses, however, GNAI3 begins to accumulate at row 1 tips, reaching a peak during early stage IV."*

Line 824" Towards the end of stage III, however, GNAI3 begins to accumulate at row 1 tips, reaching a peak during early stage IV".

Same comment as for Line 427; does not seem correct in light of the graph, where GNAI3 is steadily raising and high in stage III already.

See above edit.

Line 907:"The row 1 complex reassembles during stage IV in a stepwise manner"

Same comment, there is no clear evidence for disassembly in stage III.

We edited the sentence to: *"The row 1 complex assembles during stages III and IV in a stepwise manner, with the level of accumulation dependent on the expression of transduction proteins."*

**B4) Assuming GPSM2-GNAI3 presence at tips depends on TMIE (and TMC1/2)**

Available evidence suggest that GPSM2 and GNAI3 require TMIE (and TMC1/2) to be durably maintained, and not enriched, at row 1 tips. This manuscript actually shows a progressive decline of GPSM2 and GNAI3: for example, reduced, yet obvious enrichment for GNAI3 at P7, and further reduced enrichment at P21 in Tmie KO. The same is shown in Krey 2020, including in Tmc1; Tmc2 double KO. GPSM2 and GNAI3 are not labeled before P7 in transduction mutants in either work as far as we can tell. However, language in the manuscript suggests in several locations (below) that GPSM2-GNAI3 presence at row 1 tips requires transduction proteins, proposing that TMIE and TMCs could be transiently present at row 1 and involved.

While we agree with the reviewer that we did not sufficiently accurately describe the dependence of GPSM2/GNAI3 on the transduction complex, we do not agree with the reviewer's conclusion that the transduction complex maintains a level of GPSM2/GNAI3. We agree that there is reduced but still obvious row 1 localization of GPSM2/GNAI3 at P7.5, but both at that time and later, GPSM2/GNAI3 is much reduced in abundance in mutants compared to controls.

More accurately, it appears that the transduction complex is required for substantial accumulation of GPSM2/GNAI3 at row 1 tips in late stage III and stage IV.

To support strict requirement, the authors would need to label GPSM2-GNAI3 at earlier postnatal stages in Tmie KO (i.e. when transduction is active and GPSM2-GNAI3 are clearly at tips already in controls, P0 base until P7). As far as we know, it cannot be ruled out that GPSM2-GNAI3 still have normal row 1 enrichment in transduction mutants at these earlier stages. The evidence of detectable GNAI3 at P7 in fact rules out strict requirement already.

We quantified GNAI3 over postnatal development in Krey et al. (2020; see Fig. 3A-B there), showing that it was nearly undetectable at row 1 tips (or elsewhere in the bundle) at P0.5. As in the present manuscript, GNAI3 increased substantially at P7.5, but the time points were too infrequent to catch the GNAI3 peak we show here.

We agree that there is not a strict requirement for the transduction complex proteins for accumulation of GPSM2/GNAI3 during postnatal development. Instead, the loss of transduction complex proteins means that GPSM2/GNAI3 is reduced during this time period—but there is no evidence that GPSM2/GNAI3 reaches a high level and then declines in transduction mutants, which would seem to be necessary for the maintenance model.

The reference cited to support the puzzling presence of transduction proteins at row 1 is Cunningham 2020. We note however that this publication does not make any claim about row distribution of the epitope-tagged TMIE and TMC1/2 fusion proteins. While images show broad (yet specific) foci across P3-P6 stereocilia shafts, these tagged proteins do not appear to be enriched at row 1 tips. In the work under review, Fig. S3 does not clarify row identity of TMC1-2 signals either.

Our point is not that TMIE and TMCs are enriched at row 1 tips, but that they do actually target to row 1 stereocilia at all (as they are not seen there later).

We do not have data that indicates where the interaction of transduction proteins and GPSM2/GNAI3 occurs.

We disagree with the reviewer’s assertion that *“…Fig S3 does not clarify row identity of TMC1-2 signals…”* While the resolution of the images is not high, clearly some of the signal is near row 1 (and row 2) tips. We added insets showing bundles in the controls showing this labeling. Future investigation is required to determine whether TMC1 and TMC2 appear at all row 1 tips during development.

In addition, we note that at P7.5, row 1 length is only significantly reduced in sh2 but not in Tmie mutants (Fig. 3K,L). This is independent evidence that proteins are row 1 are not fully dependent on transduction for function. Similarly, at P21, length reduction is greater in sh2 compared to Tmie KO.

Agreed.

Finally, as GPSM2 and GNAI3 are highly enriched at OHC row 1 tips during embryogenesis, it is unlikely that transduction proteins are required in this cell type even if they were required in IHCs. This further underscores the difficulty of outlining a universal stereocilia differentiation program (point B1).

Again, our more nuanced conclusion (motivated by the reviewer's critique) is that transduction proteins are required for the stage III-IV build-up of GPSM2/GNAI3.

The following excellent sentence in the discussion should be the standard for modifications in the rest of the manuscript:

"In TmieKO/KO mutants, members of the row 1 complex are still present, but neither GPSM2 nor GNAI3 traffic efficiently to row 1 tips in the absence of transduction proteins, at least during stage IV."

Locations where these comments apply:

Line 71: "Although row 1 lacks transduction [22], in transduction mutants, GNAI3 and GPSM2 do not accumulate substantially at row 1 tips in transduction mutants [6]."

As GPSM2-GNAI have been reported at OHC tips during embryogenesis already, and were not investigated before P7.5 in this work or in Krey 2020, it would be more accurate to state that they are not maintained normally at tips in these mutants.

That's not quite accurate either. GPSM2/GNAI3 accumulate substantially during postnatal development; both the present study and the previous one show that. To say that they are "not maintained" would suggest that they reached a high concentration, then decreased in concentration. There is no evidence for this pattern in IHCs, and the evidence in OHCs is weak without developmental quantitation. (We agree that GPSM2/GNAI3 are higher at row 1 tips in OHCs than IHCs in embryonic IHCs.)

To soften the quoted sentence, we rewrote it to say: "*Although row 1 lacks transduction (22), in transduction mutants, GNAI3 and GPSM2 do not accumulate substantially at row 1 tips* ***over postnatal development*** *(6).*"

Line 446: " These results contrasted with those from TmieKO/KO IHCs, where GPSM2 and GNAI3 largely disappeared from hair bundles (Fig 10D, L);"

This fails to capture the fact that loss of enrichment is progressive with GNAI3 still well visible at P7. 5 for example. So maintenance is affected, specifically at least from the end of stage III.

Again, simple maintenance cannot explain the results either, as the intensity at P7.5 in Tmie^KO/KO (Fig. 10D) is much less than in the Tmie^KO/+ controls (Fig. 10C). We think it's fair to say that loss of transduction reduces the accumulation of GPSM2/GNAI3 that occurs during stages III-IV, but it is not fair to say that loss of transduction simply prevents maintenance of a high level.

To be more accurate in our description, however, we edited this sentence to read: *"These results contrasted with those from Tmie^KO/KO^ IHCs (Fig 10D, L), where GPSM2 and GNAI3 never reached levels seen in Tmie^KO^/+ controls (Fig 10C, K)…"*

Line 448: “; WHRN intensity was reduced, but remained most abundant in row 1 (Fig 10H).”

In fact, WHRN at P7.5 has the same profile as GNAI3 which is instead described as largely missing in several places in the text: WHRN is reduced but still present at row 1 tips. The accurate description for WHRN should apply to GNAI3 as well. Summarizing GNAI3 as instead largely missing appears to be a bias.

We agree that our descriptions are not balanced. See above edits.

Line 520: (referring to reduced row 1 proteins in Tmie but not tip-link mutants): "These results confirm that TMIE (and TMC1/2) affect distribution of GNAI3, GPSM2 and WHRN directly, not via transduction-mediated ion flux [6].

Also Abstract Line 33:"These results reinforced the suggestion that the transduction proteins themselves facilitate localization of proteins in the row 1 complex."

Also Discussion Line 740:"Our results here confirm our previous suggestion that transduction proteins themselves regulate the amount of GNAI3 and GPSM2 that accumulate at row 1 tips during stage IV [6]."

We are confused as to why this is not a new conclusion in this current study. Krey 2020 does not reach the conclusion that GPSM2-GNAI3 and WHRN do not depend on Ca++ or transduction. In Krey 2020, the possibility that transduction proteins themselves are needed is acknowledged, but results are largely interpreted and discussed as linked to Ca++. It would in fact be beneficial, and as far as we can tell, fair, to claim novelty for this important result here.

The reviewer is making a very good point here. To be honest, we had the preliminary tip-link mutant results at the time of Krey et al. (2020) but we were a long way away from being able to incorporate them into that paper at that time. While the tip-link mutant results do lead to the "transduction proteins themselves" conclusion, we were careful not to draw that conclusion in Krey et al. (2020) as that manuscript did not contain the necessary data. We made an error when writing the present paper in forgetting that these are indeed **new** results.

The indicated sentences were changed to read:

*"These results* ***showed*** *that TMIE (and TMC1/2) affected distribution of GNAI3, GPSM2 and WHRN directly, not via mechanotransduction-mediated ion flux."*

*"These results* ***showed*** *that the transduction proteins themselves facilitate localization of proteins in the row 1 complex."*

*"Our results here indicate that transduction proteins themselves regulate the amount of GNAI3 and GPSM2 that accumulate at row 1 tips during stage IV."*

Line 521: "TMC2 still targeted to stereocilia in Pcdh15av3J/av3J 522 mutants, but TMC1 disappeared (S3 Fig), suggesting that TMC2 may be sufficient for localization of GPSM2, GNAI3, and WHRN to row 1 tips.."

The conclusion here does not seem valid. In Krey 2020, the authors showed reduced but still clearly enriched GNAI3 at tips in Tmc1, 2 DKO at P7.5. Consequently, as with TMIE, GPSM2-GNAI3 are likely to rely on TMCs for maintenance rather than initial enrichment.

We agree that the conclusion is not as simple as we wrote, but we also still believe that the role of the TMCs is not simply maintenance, but in amassing a very high level of GPSM2/GNAI3.

Still, we edited the line to read: *“TMC2 still targeted to stereocilia in Pcdh15^av3J/av3J^ mutants, but TMC1 disappeared (S3 Fig), suggesting that TMC2 may be sufficient for* ***substantial accumulation*** *of GPSM2, GNAI3, and WHRN to row 1 tips.”*

Line 668:"In the absence of transduction [6] or tip links (this work), the EPS8-MYO15A complex is no longer exclusively targeted to row 1 and instead appears at all tips, also in proportion to length.

See section B2, "exclusively" is incorrect without mention of stage.

Changed to read: *"…the EPS8-MYO15A complex is no longer exclusively targeted to row 1* ***during postnatal development*** *and instead appears at all tips, also in proportion to length."*

Line 739 title "Transduction proteins localize the row 1 complex"

As mentioned above, this is an overstatement. Maintenance appears more likely.

Changed to: ***"Transduction proteins stimulate accumulation of the row 1 complex"***

Line 745: "TMIE and the TMCs do localize to row 1 near tips during early postnatal development [23]"

Line 748: "Accordingly, GNAI3 or GPSM2 may bind directly or indirectly to TMC channels localized to tips of row 1 stereocilia during development (Fig 15E).:

Line 825: "may be due to binding of GNAI3 to the complex at the tip of row 1, where it is transiently during development."

These are all overstatements. See comments above about Cunningham 2020 not discussing row identity. In particular, images do not appear to show convincing enrichment of TMIE or TMC1/2 at row 1 tips.

Line 745 is not an overstatement. The data in Cunningham et al. (2020) (see Figs. 1-2) is sufficient to say that they *"…localize to row 1 near tips…"*

Line 748 was edited to: *"Accordingly, if TMC channels transiently localize to tips of row 1 stereocilia during development, GNAI3 or GPSM2 may bind directly or indirectly to them (Fig 15E)."*

Line 825 was edited to: *"This accumulation depends on the presence of the transduction complex."*

MINOR POINTS

In abstract and throughout text, language changes between “tip-link” and “tip link”. Ex line 27 vs line 30

There is a grammatical reason for that. No hyphen is used when *“tip link”* is used as a noun, such as in *“we examined mouse mutants that eliminated tip links…”* (line 27) By contrast, we use a hyphen when *“tip-link”* is used as a compound adjective, such as in “*Use of the tip-link mutants also…*” (line 30) In this latter phrase, “*tip-link”* is the compound adjective that modifies *“mutants.”*

Abstract Line 38:"Reduced rhodamine actin labeling at row 2 stereocilia tips of tip-link and transduction mutants suggests that..."

This is only true at P7.5 (during development) while at P21 the opposite is reported, an increase (a delay?). Maybe clarify the stage? In adults, transduction appears to instead stabilizes barbed-ends.

Changed to: *“Reduced rhodamine-actin labeling at row 2 stereocilia tips of tip-link and transduction mutants* ***at P7.5*** *suggests…”*

Line 71 & 73- repeated ‘in transduction mutants’

Deleted the second *“in transduction mutants.”*

Line 79- along with [31-33] consider citing also Caberlotto PNAS 2011 where Ush1g but also Cdh23 KO is reported to reduce row 2 and 3 stereocilia length. I think this publication is not cited. It describes variably lost stereocilia, an extreme outcome possibly mirrored in this study by the reduced number of row 3 stereocilia (Fig. 2I-J).

We don’t think that the original line 79 is the right place for that citation, as it strictly refers to the constitutive global mutations in *Pcdh15* and *Cdh23*. Caberlotto et al. used a Myo15a-Cre to get recombination somewhat later in development, and some of the early phenotype is therefore missing.

That said, Caberlotto et al. is an important paper and was first to provide some evidence for the connection between transduction and stereocilia length. We tied this observation together with the loss of CAPZB when transduction disappears to suggest that the loss of row 2 and 3 stereocilia in the Caberlotto paper (ref. 95) is due to the loss of CAPZB at tips:

*“As shown here and previously (6), localization of CAPZB at stereocilia tips requires transduction. In older mutant hair cells that have lost transduction, row 3 and even row 2 stereocilia degenerate (95), which could be due to the loss of CAPZB and a subsequent loss of stereocilia actin (14).”*

Line 80 and 82 – best to define ‘membrane tenting’ first, line 82 is defined after first use line 80

Good point. We moved the first noted sentence to the end of the following paragraph, combining it with the concluding point: “Because tip links gate transduction channels (38), Cdh23 and Pcdh15 mutants lose both transduction and membrane tenting (37), and shorter stereocilia in homozygous Cdh23 and Pcdh15 mutants have rounded tips instead of beveled ones (37).”

Line 84: "Tension in tip links thus promotes actin polymerization in the shorter stereocilia rows, independent of channel gating [37]."

Is this in fact experimentally verified? When tenting/tip-links are removed, MET channels are no longer gated, so how can the specific role of tension be assessed? Caberlotto et al. Bioarchitecture 2011 [37] is a speculative paper when it comes to how absence of Usher1 proteins leads to reduced row 2 and 3.

Fair point. The mechanism has been experimentally demonstrated for other actin-membrane systems (actin polymerization is enhanced when the membrane is moved away from the growing end of actin filaments), but it has not been shown definitively for tip links and stereocilia.

To soften this point, we changed the sentence in question to read: *“These results suggest the possibility that tension in tip links promotes actin polymerization in the shorter stereocilia rows, independent of channel gating (41).”*

Comment on the Introduction: given the interesting results obtained in this study, the Introduction should probably remind readers that "tip-link" proteins form ubiquitous transient shaft links, and kinocilial links as well. As concluded by the authors, these links probably explain the bulk of stereocilia defects reported in Cdh23 and Pcdh15 mutants.

The following sentence was included in the introductory remarks on tip links and the cadherins: *“CDH23 and PCDH15 contribute to transient lateral links (24, 26, 31, 32), which interconnect stereocilia along their shafts during development, and kinocilial links (33, 34), which connect several central row 1 stereocilium to the axonemal kinocilium.”*

Line 156 – It might be useful in this text section to explicitly define what phase III and IV correspond to exactly.

Changed the first sentence to read: *“Tilney suggested that hair-bundle development in chickens could be divided into four stages, where stage I hair cells have apical surfaces with equal-length microvilli, stereocilia in stage II cells begin to lengthen and form the staircase, stage III stereocilia widen without lengthening, and stage IV stereocilia undergo final lengthening (1).”*

Fig S1. Both panels E and F are labelled P7.5, is this correct?

Those are two different possibilities for stereocilia arrangement at P7.5. While one might think that the bundle looks like that in S1E, given that later the stereocilia are at equal length, parsing the data of S1A and S1B suggests that the bundle looks like that in S1F. The text of the figure legend states: *“A peripheral-to-central gradient of stereocilia length for row 1 and row 2 was seen at P7.5 (F; red gradient symbol), rather than uniform lengths (E; red line). Stereocilia lengths largely equalized by P21.5 (G).”*

Fig. 6 – It would be useful to have the genes indicated as caption in (A), as in previous figures. Het; Het or Het; KO is not clear when browsing figures. The full genotype was actually indicated in similar circumstances in Fig. S5.

Agreed and now corrected to include full genotype description.

Fig. S4 C, D: WHRN caption in merge but GNAI3 in single channel. Same issue in all the right column panels.

Corrected for panels C-D, G-H, and K-L.

Line 283: figure citation appears incorrect. (Fig 4Gi-Fi) should be Fig 4Ai – Fi ? and (Fig 4Aii-Dii) should be Fig 4Ai-Di talking about width not CV.

The reviewer is right about the first figure citation (corrected as indicated). The second citation is correct, as we were talking about width variability. To clarify, we changed *“width variability”* in the second sentence to *“width CV.”*

Lines 292-294 wrong panels referenced in figure legend, there is no I-P in Figure 4

Legend has been corrected.

Line 296: what would be a systematic variation in length within a row? how would it be distinguished from stochastic? Or, how do results in Fig. 5 allow to decide for stochastic? If CV is high for both controls and mutants it is not the base of this distinction.

Imagine three examples of a single stereocilia row with six stereocilia:

1. Sequentially 6, 6, 6, 6, 6, and 6 µm

2. Sequentially 5, 6, 7, 7, 6, and 5 µm (systematic variation)

3. Sequentially 4, 8, 8, 4, 6, and 6 µm (stochastic variation)

The mean stereocilia length and CV for these three examples are:

1. Mean = 6.00, CV = 0

2. Mean = 6.00, CV = 0.15

3. Mean = 6.00, CV = 0.30

Thus the CV indicates variability for the second two examples. As the quoted text indicates, the variation could be systematic or stochastic. If we then determine the adjacent stereocilia length differences, we get:

1. Sequentially 0, 0, 0, 0, and 0 µm

2. Sequentially 1, 1, 0, 1, and 1 µm

3. Sequentially 4, 0, 4, 2, and 0 µm

The mean Δ stereocilia length (the adjacent difference) and CV for these three examples are:

1. Mean = 0, CV = 0

2. Mean = 0.80, CV = 0.56

3. Mean = 2.00, CV = 1.00

Thus a high mean of the Δ stereocilia length is more consistent with the stochastic example. This illustration shows why just looking at the mean and CV of the stereocilia lengths isn't sufficient; the deeper analysis of Δ stereocilia length allows the variability to be highlighted.

The text is accurate; the full appropriate quote reads: *"An elevated CV for IHC stereocilia length for tip-link mutants (Fig 3) could arise from systematic variation in length within a row or from stochastic variation within the row. To probe the source of high CV, we used Imaris surfaces to measure the differences in length between adjacent (side-by-side) stereocilia of row 1 or row 2 …"*

It is important to note that we indicate that we are probing the source of high CV, not deterministically deciding whether it is from systematic or stochastic variation.

Reviewer #1 is also concerned about our "convoluted" method of analysis. We believe, however, that the incredibly rich dataset we have allows us to dig deeper into what is going on and pull out interesting observations.

Line 355 Fig 6D-E should be Fig6D, F, etc. In general, correct panel labels for Figure 6 descriptions in Results main text.

Corrected.

Line 443 “heterozygotes from mutant IHCs” meaning is unclear

That was confusing, we agree! Changed to: *“As in C57BL/6 stereocilia, in heterozygotes from* ***mutant mouse lines****, GNAI3, GPSM2, and EPS8 were concentrated at tips of row 1 stereocilia* ***from IHCs*** *(Fig 10A-D, I-L).”*

Line 454 Fig 10 I-L refers to localization of GPSM2, not GNAI3

Corrected.

Line 457 panel labels should be “…I and J, K and L”

Corrected.

Line 462 in Figure legend 10 seems redundant

Yes. We moved that line up to replace the previous line referencing the arrows.

Lines 477 & 478 error in panel labels in Figure 11 legend.

Corrected.

Line 567: "In Pcdh15av3J567 /+ controls, the high level of row 2 labeling by rhodamine-actin seen in control bundles at P7.5 (Fig 13A, M) had disappeared by P21.5 (Fig 13G, P);"

This sentence is a little confusing. It seems it could apply to all controls and not just Pcdh15 controls (i.e. free barbed ends are drastically lost at row 2 at P21.5 compared to P7.5).

Yes, that was confusing. We cleaned up the figure references and lumped Pcdh15 and Tmie mutants together.

Line 580: "By contrast, in Myo15ash2/sh2 mice, row 1 and row 2 rhodamine-actin labeling was elevated to the intensity level seen in row 1 from control mice at P7.5 (Fig 13E-F, O)".

This is confusing; should the sentence say "..elevated to the intensity level seen in row 2 from control mice at .."? It seems clear from images and graphs that sh2 mutants at P7 have a reduced accumulation at row 2 but increased at row 1 (uniform by row).

Yes, the reviewer is correct. The sentence was corrected as indicated (row 2 from control not row 1).

In this figure, it is surprising to see how modest the raise in Rhod-actin signal is at P21 in the mutants compared to the control on the graphs. Visually, signal appears to be absent in controls and massively increased in mutants. This is probably linked to the Y axis range adopted for much higher signals in earlier controls at row 2?

Yes, the difference between the het and KO values at P21.5 do not appear as large in the graph as they do in the images, and yes, this is due to the y axis range used. If the range was adjusted, the difference between the het and KO values would be appear to be much larger.

Line 528-529 Figure 12 legend: Cdh23 mutants not shown in Figure 12. Error in panel labels and lack of GPSM2 mention in line 531. Legend missing for M-P

Fig. 12 legend is corrected (multiple errors).

Line 624: Error in panel labelling. Fig 15D is EPS8, E is GPSM2, F capping proteins

Corrected (see reviewer #1 comments on this figure).

Line 667, 679: the reference for MYO15A-EPS8 at all tips and independent GNAI3 there is Tadenev 2019 and not Tarchini 2016 [7].

Corrected.

Line 703: Absent kinocilial links could well explain part of the defects as well, especially the lack of packed distribution. Besides adding links to the Introduction (see above), this part of the Discussion could be broadened to discuss kinocilial links as well.

We have added comments on kinocilial links to the Discussion, as suggested.

Line 755:"during stage III, row 2 stereocilia shorten when row 1 lengthens,.."

This is confusing, as here stage III row 1 stereocilia were shown to be very stable and not to lengthen (Fig. 1G). Or should this sentence be about stage IV, not III ?

Yes, that was an error. Plus that sentence tried to have too many ideas in it. Changed to: *“Transduction controls actin-regulatory mechanisms in row 2 differently than those in row 1. For example, during stage III, row 2 stereocilia shorten while those of row 1 are static. In addition, the complement of proteins at row 2 tips differs substantially from that at row 1 tips.”*

Line 787: The Discussion at this point appears to bring back earlier discussion points and show some degree of redundancy, as if two drafts were fused at this position without fully consolidating by topic. Possibly a similar comment starting at Line 863, "How the mutant phenotypes arise".

The Discussion was organized into three parts: (a) discussion of several individual observations, (b) overall model for length regulation (***"A model for hair-bundle length regulation during stages III and IV"***), and (c) an explanation of the mutant phenotypes in the context of that model (***"How the mutant phenotypes arise"***).

There is some redundancy, but we felt it was indeed important to bring back the earlier discussion points to either explain the model or explain the phenotypes.

Related: two ideas are presented in two different locations for a potential binding between GPSM2-GNAI3 and the transduction apparatus:

Line 749: "This coupling could be mediated by the established CIB2-WHRN interaction [85];"

Line 825: "may be due to binding of GNAI3 to the complex at the tip of row 1, where it is transiently during development."

These sentences are not in conflict; the line 825 sentence simply suggests some sort of interaction with the transduction complex, whereas the line 749 sentence offers a possible specific interaction. CIB2 is definitively part of the transduction complex (see the Jeong structural paper, for example). We are not suggesting that GPSM2/GNAI3 has to bind to the pore-containing subunits, but rather to one of the complex members that goes away when components of the complex are knocked out (e.g., CIB2 goes away).

Reviewer #3: General comments

Krey et al. provide a comprehensive description of how cochlear inner hair cell (IHC) bundles acquire their stereotypical shape: 3 rows in an uneven stair-step arrangement of tightly-regulated heights. They probe developmental regulatory mechanism with several types of mutants: transduction mutants affecting molecules that belong to the mechanoelectrical transduction apparatus at the tips of rows 2 and 3; tip link mutants in cadherins that connect the transducing tips of rows 2 and 3 to taller stereocilia in rows 1 and 2, respectively; mutations in G-protein-related proteins that congregate at stereociliary tips; and mutants affecting actin-capping and -severing proteins.

The data are quite beautiful and the writing is a model of clarity, especially given the quantity of data being presented. Setting up the staircase of stereocilia depends in complex ways on both the groups of proteins just mentioned and on developmental time, with the hair bundle passing through a series of stages to reach the mature configuration. Krey et al. lay out, in considerable but not excessive detail, the morphological changes in control IHCs and the various mutant IHCs as functions of time and hair bundle stage, and pull the data together in a summary model, graphically illustrated in Fig. 15. At several stages - especially Introduction, Discussion and Conclusions - they summarize the significance of the new observations, better than I will do here.

The work leverages the mammalian hair bundle for its exceptional value as a model for cellular structure-function studies. Building on previous work (well-summarized), the authors offer a number of new insights - notably, separating the accumulation of stereocilia 'building blocks" from their regulation and isolating structural development roles for molecules already known to be necessary for gating and expressing mechanotransduction. Along the way, new information addresses previous arguments in the literature, specifically indicating that Tmie, part of the met channel complex, does not interact with protocadherin 15, and that members of the row 1 tip complex do not co-assemble at once. Given the importance of hair bundle structure for filtering different kinds of mechanical stimuli, these carefully curated and detailed data invite comparative studies on the regulation of other hair bundles.

Suggestions/ questions:

1. Graphical illustrations - Figure 15 was so welcome that I wonder whether individual, smaller scale, summary diagrams at the end of each major set of experiments would help readers keep the important observations in mind (or at least this reader).

We added a new panel to Fig 1 that shows the four Tilney stages.

2. Tip links and row 3 vs row 2 asymmetries -

a. A major discussion point, tip links are only depicted in the SEMs of Fig. 2 - for the sake of non-aficionados, I suggest finding a way to include them in Fig 15.

That is an excellent point. We added a blowup of one of the stereocilia pairs in Fig 15 and added in a tip link. We also added tip links to Fig. 1O and indicated them with callouts. We indicated that only rows 2 and 3 have transduction.

b. Given the importance of tip-link tension and tip tenting to the model of hair bundle growth, what is known about how tip links compare across rows - are they of similar length and angle for rows 2 and 3?

To our knowledge, nobody has systematically compared row 1-2 and row 2-3 tip links.

c. In Fig 15 legend, transduction is said to lead to "accumulation of DSTN and CFL1 at row 2 tips, triggering…shrinkage of row 2". What is going on at row 3 - which also has transduction, but apparently no accumulation of DSTN and CFL1 ? and ends up remarkably thinner than row 2.

We agree that this is an interesting paradox. In Krey et al. (2020) we speculated that the open probability of the row 3 transduction channels may be considerably less than the row 2 channels since the presumed motor at upper end of the tip link controlling row 3 channels may be inhibited by Ca2+ entering row 2 tips (see Figure S7C in Krey et al., 2020).

We also note that McGrath et al. (2021) show elevated free barbed end levels not only in row 2, but also in row 3 (Fig. 2F,G in that paper). One can also see some ADF/CFL at row 3 tips in their Figs. 4 and 5 as well. ADF/CFL is not as enriched at row 3 tips as in row 2 tips, but this seems likely to be due to the lower cross-sectional area of row 3 tips, which will affect the measurement.

Detailed comments

Line 377 - I did not find row 3 stereocilia obvious in the control bundles in Fig. 7A-D - label?

To improve their visibility, we both increased the gain on the images to bring up the shorter stereocilia (saturating rows 1 and 2), and added yellow boxes to highlight their positions.

Line 873 - doesn't scan - "…slowly pruning the shorter rows"?

Yes, that sentence was messed up. Changed to: *“Capping proteins that usually enrich at row 2 (EPS8L2, CAPZB, TWF2) are present at all rows, presumably preventing pruning of the shorter rows.”* (The original comment about stereocilia thickness was not relevant here.)

Fig. 15 - Legend does not match figure - looks as though panel C was added late. Apparently it is meant to show the time course of tip link and transduction appearance, but best to make that clear by showing the stages underneath, as

The reviewer is correct. We added: *“****C****, Timing of tip-link and transduction appearance relative to stages depicted in panels D-F.”* And we corrected the label references for panels D-F.
